# Utilization of the Shensheng-Piwen changed medicinal powder extracts combines metal-organic frameworks as an antibacterial agent

**DOI:** 10.3389/fcimb.2024.1376312

**Published:** 2024-06-07

**Authors:** Haiqun Jin, Xiujun Zhang, Xiaoqing Ma, Xin Meng, Zhenguang Lin, Xiaoyuan Li, Guojie Hu, Yao Chen

**Affiliations:** ^1^ Department of Traditional Chinese Medicine, Affiliated Hospital of Qingdao University, Qingdao, China; ^2^ State Key Laboratory of Medicinal Chemical Biology, Nankai University, Haihe Laboratory of Synthetic Biology, Tianjin, China; ^3^ Tianjin Academy of Traditional Chinese Medicine Affiliated Hospital, Tianjin, China; ^4^ Key Laboratory of Marine Drugs, Ministry of Education, School of Medicine and Pharmacy, Ocean University of China, Marine Biomedical Research Institute of Qingdao, Qingdao, China; ^5^ College of Materials Science and Engineering, Qingdao University, Qingdao, China; ^6^ Key Laboratory of Industrial Microbiology, Ministry of Education, College of Biotechnology, Tianjin University of Science and Technology, Tianjin, China; ^7^ Shandong Academy of Pharmaceutical Sciences, Jinan, China

**Keywords:** Shensheng-Piwen changed medicinal powder, antimicrobial, biofilms, metabolomics, synergy, infections

## Abstract

**Introduction:**

Widespread opportunistic pathogens pose a serious threat to global health, particularly in susceptible hospital populations. The escalating crisis of antibiotic resistance highlights the urgent need for novel antibacterial agents and alternative treatment approaches. Traditional Chinese Medicine (TCM) and its compounds have deep roots in the treatment of infectious diseases. It has a variety of active ingredients and multi-target properties, opening up new avenues for the discovery and development of antimicrobial drugs.

**Methods:**

This study focuses on assessing the efficacy of the Shensheng-Piwen changed medicinal powder (SPC) extracts against opportunistic pathogen infections by broth microdilution and agar disc diffusion methods. Additionally, biofilm inhibition and eradication assays were performed to evaluate the antibiofilm effects of SPC extracts.

**Results:**

Metabolite profiles were analyzed by LC-MS. Furthermore, the potential synergistic effect between SPC and Metal-Organic Framework (MOF) was investigated by bacterial growth curve analysis. The results indicated that the SPC extracts exhibited antibacterial activity against *S. aureus*, with a minimum inhibitory concentration (MIC) of 7.8 mg/mL (crude drug concentration). Notably, at 1/2 MIC, the SPC extracts significantly inhibited biofilm formation, with over 80% inhibition, which was critical in tackling chronic and hospital-acquired infections. Metabolomic analysis of *S. aureus* revealed that SPC extracts induced a notable reduction in the levels of various metabolites, including L-proline, L-asparagine. This suggested that the SPC extracts could interfere with the metabolism of *S. aureus*. Meanwhile, the growth curve experiment proved that SPC extracts and MOFs had a synergistic antibacterial effect.

**Discussion:**

In conclusion, the present study highlights the potential of SPC extracts as a novel antibacterial agent against *S. aureus* infections, with promising biofilm inhibition properties. The observed synergistic effect between SPC extracts and MOFs further supports the exploration of this combination as an alternative treatment approach.

## 1 Introduction

Bacteria are omnipresent on Earth, and among them, opportunistic pathogenic bacteria play a crucial role in human health (Kwiecinski and Horswill, 2020; Mayer et al., 2023). Since the discovery of antibiotics, antimicrobial resistance has emerged as a global challenge, with millions of people dying each year from opportunistic or primary infections. For example, bacterial vaginosis (BV), caused by vaginal dysbiosis in women of childbearing age, has a prevalence of 29% in the United States. Due to increasing microbial resistance to BV pathogens, common drugs such as metronidazole and clindamycin have shown decreasing cure rates for BV (Muñoz-Barreno et al., 2021). The decreasing efficacy of antibiotics and the lack of new drugs against resistant organisms continually emphasize the importance of developing novel antibacterial agent and new antibacterial alternative therapies. In addition, with the emergence of multidrug-resistant bacteria, hospital-acquired infections have become a serious public health problem. It is estimated that approximately 25% of hospitalized patients in developing countries are affected by nosocomial infections (Lemiech-Mirowska et al., 2021). Understanding the mechanisms by which bacteria develop resistance is therefore essential to tackling the problem of antimicrobial resistance. Resistance-associated mutations, evolution and horizontal gene transfer in bacteria are the main reasons for the appearance of multi-drug resistant bacteria (Machado et al., 2023). For example, methicillin-resistant *Staphylococcus aureus* (*S. aureus*) contains the methicillin resistance gene (mecA), which has spread globally, increasing the healthcare burden in many countries (Lee et al., 2018); vancomycin-resistant *Enterococcus faecalis* contains the vancomycin resistance gene (vanA), which is recognized by WHO as one of the antimicrobial-resistant pathogens posing a major threat to public health (Dos Santos et al., 2020). Besides, microbial biofilms are a form of protection for microorganisms against external influences. Extracellular matrix and various microbial colonies combine to form biofilms that can adhere to biotic or abiotic surfaces. It is well known that almost all bacteria and some fungi have the ability to form biofilms, which enables them to evade the host immune response and tolerate various antimicrobial drugs (Cangui-Panchi et al., 2022). *S. aureus*, a potent biofilm producer, is the most common causative agent in global infections. The virulence factors produced by *S. aureus* allow them to spread and become pathogens under favorable conditions, and its biofilm formation can also modulate the expression of virulence factors to resist recognition or phagocytosis by the innate immune system. Similarly, virulence factors produced by different pathogenic *Escherichia coli (E. coli)* contribute to the formation of biofilms with different adherence structures, allowing them to persist at the site of infection and greatly enhancing bacterial drug resistance (Cangui-Panchi et al., 2023).

To address the challenge of opportunistic pathogens, it is vital to continue researching and identifying new antibacterial agents and alternative treatment strategies. This involves exploring novel antibacterial compounds, understanding the mechanisms of bacterial resistance, and developing innovative approaches to combat opportunistic pathogens (Moellering, 2011; Bhardwaj et al., 2022). Traditional Chinese medicine (TCM) is an integral part of complementary and alternative medicine (CAM). Traditional Chinese medicine compounds (TCMCs) are combinations of herbal medicines derived from plants found in nature (Su et al., 2020; Song et al., 2022). TCM has been used for disease prevention and treatment in China and East Asia for thousands of years (Zhang et al., 2019). It is well known that TCM prescriptions, which originates from long-term practical experience and traditional medical theories, is essentially various combinations of different herbs based on specific dosages (Zhao et al., 2021). A large number of studies have been reported on TCM prescriptions in terms of antimicrobial activity, enhancement of body immunity and regulation of intestinal flora. For example, Maxing Shigan Decoction is an effective treatment for pneumonia caused by *Pseudomonas aeruginosa* (Xu et al., 2024). Sanhuang Xiexin decoction can regulate intestinal flora to improve colitis in mice (Wu et al., 2022). According to historical records in the book “Ancient and Modern Medicine” written in 1589 AD, a classic TCMC prescription called Shensheng-Piwen changed medicinal powder (SPC) was used during outbreaks of infectious diseases. This prescription contains ten types of TCM, such as *Atractylodes lancea*, *Angelica dahurica and Cyperus rotundus L*. Many of these herbs have been reported to have numerous biological effects. For example, essential oils extracted from *Cyperus rotundus L.* have antimicrobial activity against foodborne pathogens (Hu et al., 2017). Therefore, we hypothesized that SPC extracts could be a novel antibacterial agent against opportunistic pathogens. Furthermore, we added Metal-Organic Frameworks (MOFs) to the extracts to broaden the application of SPC extracts. MOFs are porous crystalline polymers formed by the self-assembly of metal centers with organic ligands and numerous studies have explored their potential in antibacterial applications (Yang and Yang, 2020). ZIF-8, MIL-101(Fe), MIL-100(Fe) and HKUST-1(Cu) are among the most extensively studied MOFs (Caamaño et al., 2022; Shateran et al., 2022; Arshadi Edlo and Akhbari, 2023; Fang et al., 2024). They can exert antibacterial effects through the release of metal ions, drug loading and intrinsic properties (light response, pH response) (Zhang et al., 2022). ZIF-8, MIL-101(Fe), and MIL-100(Fe) were chosen as additives for SPC extracts due to their ease of synthesis, biocompatibility, and potential synergistic effects that may exist.

In light of this, the active ingredients of SPC were extracted by ethanol distillation. *S. aureus* and *E. coli* were selected as two representatives of opportunistic pathogens. Antibacterial susceptibility testing was performed using appropriate methods to evaluate the effectiveness of the SPC extracts against these bacteria. Additionally, different MOFs were added to the SPC extracts to investigate their compatibility. The efficacy of various formulations was compared, highlighting the potential applications of SPC and SPC-MOF combinations in combating infectious diseases.

## 2 Materials and methods

### 2.1 Main reagents and instruments

The SPC herbal decoction was prepared by the Pharmacy Department of the Affiliated Hospital of Qingdao University. Herbs were purchased from Linqu Pharmaceutical LTD, Ltd., Co.; ShangPharma Holdings Qingdao LTD, Ltd., Co. and Qingdao Shanda Tianyuan Chinese Medicine slices LTD, Ltd., Co. The prescribed dosages of SPC are shown in **Supplementary Table S1**. Quality control of SPC was carried out by UPLC-QE- Orbitrap-MS (**Supplementary Figure S1**).

Main reagents: Ferric chloride hexahydrate (FeCl_3_·6H_2_O, 98%) and 2-methylimidazole were acquired from Aladdin (Shanghai, China). Benzene-1,4-dicarboxylic acid (H_2_BDC, 99%), trimesic acid (H3BTC,98%), ferric nitrate nonahydrate (Fe(NO_3_)_3_·9H_2_O, 98.5%), zinc nitrate hexahydrate (Zn(NO_3_)_2_·6H_2_O, AR) were purchased from Heowns. Acetonitrile and methanol were chromatographically pure, Merck, USA; formic acid was chromatographic alcohol, Thermo Fisher, USA. All solvents and reactants obtained were of analytical grade and used directly without special treatment. Deionized water was used in preparation of various solutions throughout the experiment.

Main instruments: Thermo Scientific Q-Exactive Orbitrap system, Waters Acquity H class type high performance liquid chromatograph, Waters Corporation, USA; Thermo Q Exactive Orbitrap type mass spectrometer with Nano ion source, Xcalibur mass spectrometry workstation, Waters Corporation, USA Thermo Scientific; MS105DU 1-in-100,000 balance, Mettler-Toledo Ltd., Switzerland; Powder X-ray diffraction (PXRD) measurements were recorded on a D/Max-2500 X-ray diffractometer by depositing powder on the glass substrate, 2θ from 2° to 40° with 0.02° increment. The scanning electron microscopy (SEM, HITACHI SU3500) and transmission electron microscopy (TEM, FEI Talos F200X G2) were used to analyze the morphologies.

### 2.2 Preparation of the SPC extracts

5 g of each herb was weighed, giving a total of 50 g. The herbs were crushed into granules using a high-speed Chinese medicine crusher and passed through a 40 mesh Chinese medicine sieve. Each batch of fresh herbs was extracted three times by continuous dynamic countercurrent extraction. The procedure was as follows: fresh herbs and solvent (5 times 50% ethanol) were placed in the extraction tank for the first extraction, and the extracts was collected after 2.5 hours. Then the solvent (5 times 75% ethanol) was added to the first extraction and the second extraction was carried out, and the extracts was collected after 2 hours. Finally, the second extract was mixed with solvent (5 times 90% ethanol) and the third extracts was collected after 1 hour of extraction. The extracts are filtered, concentrated and dried, and the paste percentage of the drug is determined. No separation was carried out to avoid loss of active ingredients.

The above three extracts were combined and kept in a refrigerator at 4 °C for 24 h (total 750mL). The supernatant is obtained by centrifugation at 10000 r/min for 20 min at room temperature and filtered through 0.45 μm hollow fiber membrane. The filtrate was collected and divided equally into 4 portions. Each portion contained 12.5 g of raw herbs. Take appropriate amount of chitosan powder, add 1% glacial acetic acid to prepare 1% chitosan solution, ready for use. The alcohol extracts are concentrated under vacuum until the ratio of the amount of raw herbs (g) to the volume of drug solution (mL) is 1:8. Then it is placed on a 50 °C constant temperature magnetic stirrer and stirred for 10 min. Chitosan solution (1.6 mL for every 1 g of raw herbs) is added to the alcoholic extracts and continue stirring for 1 min. Finally the solution was taken out and left at room temperature for 12 h. Observe and record the clarity of the solution and the shape of the precipitate.

### 2.3 Preparation and characterization of MOF

MIL-101(Fe) (Peng et al., 2022), MIL-100(Fe) (Fang et al., 2018), and ZIF-8(Zn) (Wang et al., 2023) were prepared according to the reported procedures in the literatures.

MIL-101(Fe) was synthesized from a mixture of 0.68 g (2.5 mmol) FeCl_3_·6H_2_O and 0.21 g (1.25 mmol) H_2_BDC in 15 mL DMF. The suspension was sonicated to complete dissolution and then was sealed into a Teflon-lined stainless steel autoclave and heated at 110°C for 24 h. The orange solid was obtained by centrifugation and washed by DMF and EtOH for three times to remove the unreacted raw materials. Finally, the product was dried overnight at 70 °C.

MIL-100(Fe) was synthesized from a mixture of 2.4 g (6 mmol) Fe(NO_3_)_3_·9H_2_O and 0.84 g (4 mmol) H_3_BDC in 6 mL deionized water. The mixture was stirred at room temperature for 1 h and then was heated at 160°C for 12 h. The solid product was centrifuged and washed with deionized water and EtOH for three times for purification. Finally, the resultant MIL-100(Fe) was dried overnight at 90 °C.

ZIF-8 Zn(NO_3_)_2_·6H_2_O (2.0 g) was added into 40 mL methanol and stirred until it was completely dissolved. Next, 2-methylimidazole (2.0 g) was added to the above solutions and stirred. Then a white solid was obtained after stirring for two hours and leaving overnight at 60 °C. The final ZIF-8 was made by drying and washing it three times with methanol.

### 2.4 Bacteria and culture conditions

Gram-negative *E. coli* ATCC 11229 and gram-positive *S. aureus* ATCC 6538 were selected as model microorganisms to evaluate the antimicrobial activities. Bacteria were incubated overnight at 37 °C 220 rpm. The bacterial concentration was then adjusted to ~10^6 CFU/mL using LB medium for subsequent experiments. Bacterial concentration is usually determined by the optical density of the bacteria suspensions at 600 nm (OD600 nm).

### 2.5 Metabolomic analysis

*S. aureus* in growth phase (OD600 = 0.6) was co-incubated with MIC under SPC for 6 h. The untreated group served as a control. Bacteria were harvested at 10,000 rpm and used as samples for metabolomics analysis. First, 10 mg solid sample were accurately weighed, and the metabolites were extracted using 80 µL methanol: water (4:1, v/v) solution. The mixture was allowed to settle at -10 °C and treated by high throughput tissue crusher at 50 Hz and followed by ultrasound at 40 kHz. After centrifugation at 13000 g, the supernatant was transferred to sample vials for analysis. Ultra-performance liquid chromatography - mass spectrometry (UPLC-MS) analysis was performed by Majorbio Bio-Pharm Technology (Shanghai, China). Chromatographic column: Infinity Lab Poroshell 120EC-C18 (150×2.1mm, 2.7μm) column, mobile phase: 0.1% formic acid water (A) - acetonitrile (B); Flow rate: 0.2 mL-min^-1^; Injection volume: 2 µL; Column temperature: 35 °C; Elution method: 0~5 min, 10% B; 5~10 min, 10%-21% B; 10~20 min, 21% B; 20~30 min, 21%-70% B; 30~35 min, 70% B. Mass spectrometry conditions: positive and negative ion mode detection, sheath gas flow rate 40 psi (1 psi = 6.895 kPa), auxiliary gas flow rate 10 psi; spray voltage 3.8 kV; capillary temperature 320°C; auxiliary gas temperature 350°C; mass scan range m/z 100–1500, detection resolution 70,000 FWHM. Ionization energies 20, 30, 40 eV, resolution 17500. The samples of the control, SPC-treated, and QC groups were subjected to UPLC for component separation; a single component entered the ion source of the high vacuum mass spectrometer for ionization and was separated according to the mass-to-charge ratio (m/z) to obtain a mass spectrum. Finally, the mass spectra of the samples were analyzed. The qualitative and quantitative results of the samples were obtained. The LC-MS data from sample pellets were processed using the Majorbio Cloud Platform (Shanghai, China), and metabolites were identified. Normalized data were visualized by partial least squares-discriminant analysis (PLS-DA) using the ropls package in R. The ellipses in the PLS-DA plots were employed to characterize metabolic perturbations among groups in a Hotelling T2 region with a 95% confidence interval threshold. The variable importance in projection (VIP) was calculated based on the PLS-DA model to identify significant metabolites with a VIP >1.0 and P-value <0.05. The KEGG (http://www.kegg.com/) database was used to explore related metabolic pathways. The detailed methods can be seen in the **Supplementary Information**.

### 2.6 K–B test

The SPC and MOFs were subjected to an antimicrobial experiment using the diffusion method described by Fiebelkorn et al (Fiebelkorn et al., 2003). The K–B test, commonly known as the agar disc diffusion method, is a classical method to evaluate antibacterial activities of samples through the average inhibition diameter, which is obtained by placing antibacterial agents in an agar plate containing a certain concentration of bacterial suspension and then incubating. Typically, powders of MOFs were pressed into discs. The discs were placed firmly on the agar plates which were uniformly covered with *E. coli*. All the plates were incubated at 37°C for 24 h, following which the zone of inhibition was measured. These data represent the average of at least three different independent experiments.

### 2.7 Minimum inhibition concentration assay

The MICs of SPC were determined by a microtiter broth dilution method (Ruparelia et al., 2008). The SPC solutions were diluted in a 96-well plate with LB medium (62.5, 31.25, 15.6, 7.8, 3.9, 1.95, 0.98 mg/mL), bacteria of the same concentration (~10^6 CFU/mL, 100 μL) were added to each well and cultured in a thermostatic incubator (37 °C) for 16–18 h. The final volume of each well in the 96-well plate is 200 μL. Ampicillin was employed as positive control, bacterial grown without treatment as negative control and LB medium as blank control. MIC was measured by the optical density values at 600 nm and minimum bactericidal concentration (MBC) was calculated by standard plate count method. These data represent the average of at least three different independent experiments.

### 2.8 Morphological characterization of *S. aureus*

*S. aureus* suspension was cultured in liquid medium until OD600 = 0.5, and 2 mL of each group was centrifuged to remove the supernatant. To the experimental group, 2 mL of SPC (MIC) solution was added and incubated for 6 h at 37 °C on a shaker. LB medium was used to replace SPC extracts in the control group, and other operations were the same as the experimental group. The bacterial solution of each group was centrifuged (5500 rpm) for 12 min, and washed 3 times with PBS. Then the *S. aureus* obtained by centrifugation in each group was fixed with 2.5% glutaraldehyde at 4°C for 5 h and was dehydrated with gradient ethanol (50%, 70%, 80%, 90%, 100%) for 15min. Subsequently, the morphological changes of bacteria were observed by SEM and TEM (Garcia Maset et al., 2022).

### 2.9 Determination of bacterial growth curves

The growth curve of *E. coli* after MOFs treatment was determined using a method similar to that described in (Rasool et al., 2016). The *E. coli* suspension was resuspended to 1.0×10^6 CFU/mL, and different concentrations of MOFs were added to each group and cultured on a shaker at 37 °C. The absorbance at 600 nm was measured every 3 hours using a microplate reader. Finally, the appropriate concentration of MOF was obtained. The combined antibacterial activity of SPC and MOF can also be expressed by the bacterial growth curve. In the same way as above, SPC (final concentration of MIC) and MOF (final concentration based on screening) were added to *E. coli* solution (1.0×10^6 CFU/mL) to determine the absorbance every 3 h. PBS was added as a positive control group and culture medium group as a negative control group. These data represent the average of at least three different independent experiments.

### 2.10 Biofilm assay

#### 2.10.1 Biofilm inhibition

The methods were slightly modified according to the literature (Aiassa et al., 2016). The log phase bacterial suspension was adjusted to 1 × 10^6 CFU/mL using LB medium. 200 μL of suspension was added to each well in a 96-well plate and incubated at 37°C for different times to form biofilm. The bacteria-free group was the negative control. After incubation, the supernatant was removed and each well was washed three times with sterile PBS. Crystal violet was used as an indicator of total biofilm biomass. So crystal violet dye (100 μL) was added to each well and incubated for 30 min, and the stained biofilm was washed three times with PBS. 33% glacial acetic acid solution (200 μL) was then added to quantify the biofilm by optical density (Ommen et al., 2017). The time point at which the highest absorbance was obtained was the time at which the biofilm was fully formed.

To test the inhibitory effect of SPC on biofilm formation, *S. aureus* (200 μL, 10^6 CFU/mL) was added to 96-well plates and co-incubated with different concentrations of SPC. The group without SPC treatment was the positive control group. Crystal violet staining was performed as described above. biofilm inhibition% = ((untreated OD_595_ - treated OD_595_/untreated OD_595_) × 100. The average of three replicated 96-wells were used to conclude the quantity of biofilm formation.

#### 2.10.2 Biofilm eradication

The bacterial solution at a concentration of 1 × 10^6 CFU/mL was added to a 96-well plate at 200 μL per well. The 96-well plate was then incubated at 37 °C for 3 days to form a mature biofilm. After incubation, the supernatant was removed and each well was washed 3 times with sterile PBS. Next, different concentrations of SPC (0.5MIC, MIC, 2MIC, 3MIC) were added to each group, and the plates were incubated at 37 °C for another 24 hours. Biofilms were obtained by washing three times with sterile PBS. The LB medium group was the positive control and the bacteria-free group was the negative control. Finally, biofilm quality was assessed by crystal violet staining as described above. Biofilm eradication rate = ((untreated OD_595_ - treated OD_595_/untreated OD_595_) × 100. The average of three replicated 96-wells were used to conclude the quantity of biofilm formation.

#### 2.10.3 Morphology analysis of biofilm by confocal laser scanning microscopy

Different groups of biofilms were slightly rinsed with sterile PBS to remove unattached bacteria, and then biofilms were stained with mixed SYTO9 and PI dyes (20 μL, live/dead BacLight bacterial viability kit, 1:1) in the dark for 20 min. Afterward, stained biofilms were visualized using a Leica TCSSP8 confocal laser scanning microscope (CLSM, Leica Microsystems, Germany). Generally, dead bacteria present red fluorescence, whereas live bacteria mainly show green fluorescence. Images with size of 300 μm × 300 μm were collected (Qiu et al., 2019).

### 2.11 Statistical analysis

All experiments were repeated at least three times. Differences between groups were assessed using Student’s t-test, one-way analysis of variance (ANOVA) and Tukey’s multiple comparison test. All analyses were performed on GraphPad Prism. Data were expressed as means ± standard deviations (Mean ± SD). P< 0.05 was considered as statistical significance. ***indicates p<0.001; **indicates p<0.01; *indicates p<0.05.

## 3 Results

### 3.1 Evaluating the antibacterial performance of SPC

**Figure 1:**
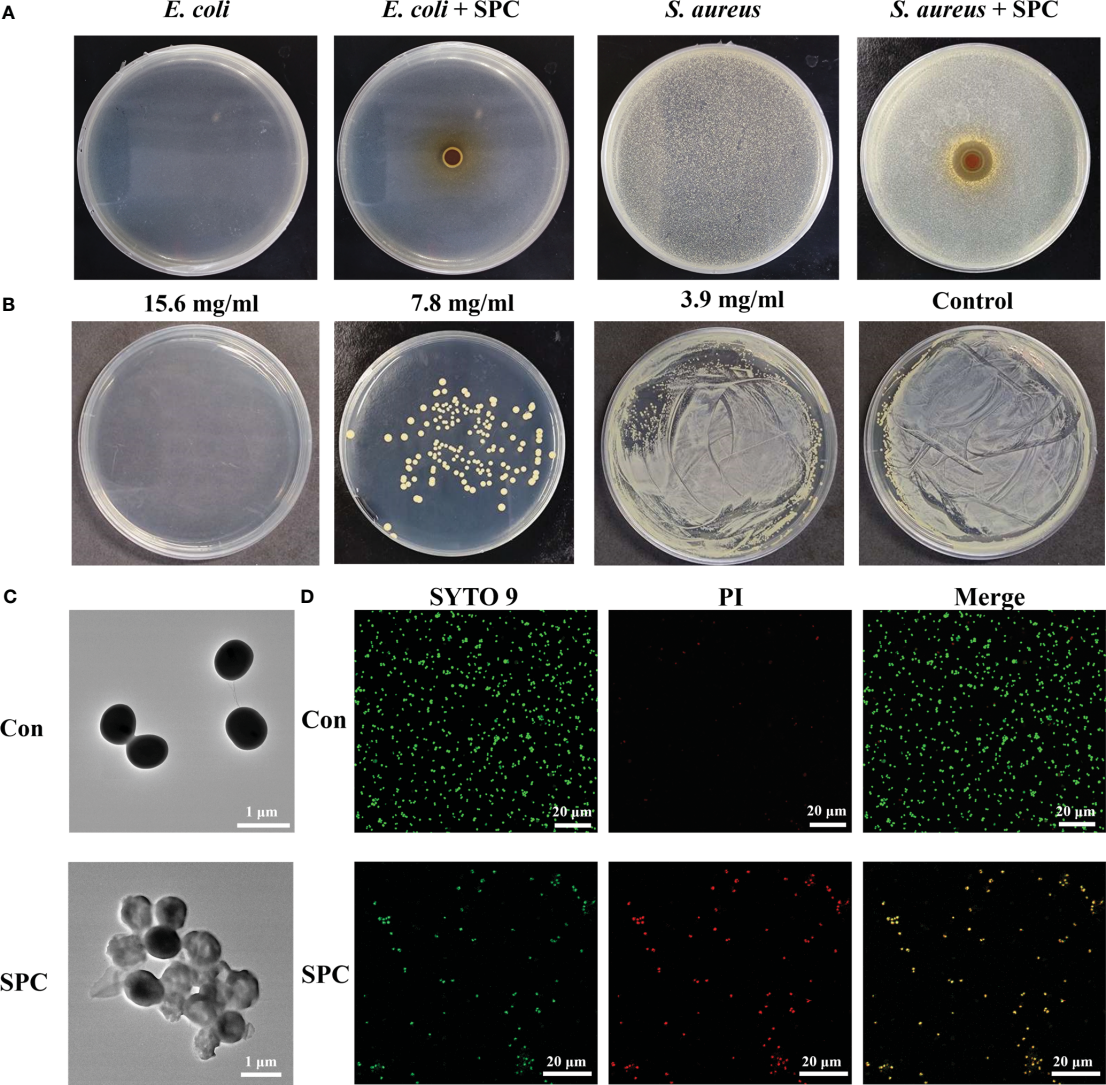
Study on antibacterial effect of SPC. **(A)** Representative image of inhibition zone of SPC for *E. coli* and *S. Aureus* (n=3 per group). **(B)** Amount of live bacteria of *S. aureus* treated with different concentrations of SPC (n=3 per group). **(C)** Representative TEM image of *S. Aureus* exposed and unexposed by SPC (n=3 per group). Scale bar: 1 μm. **(D)** CLSM of live/dead staining after different treatments (n=3 per group). Scale bar: 20 μm.

According to previous research methods, the antibacterial activity of Gram-negative strains of *E. coli* and Gram-positive strains of *S. aureus* was determined using the disk diffusion method. As shown in **Figure 1A**, the SPC extracts produced an inhibition zone (clear areas with no bacterial growth) against *S. aureus* but had little effect on inhibiting the growth of *E. coli*. This suggests that SPC primarily inhibits the proliferation of *S. aureus*. To gain deeper insights into the biological effects of SPC, we conducted antibacterial experiments using the broth microdilution method and standard plate counting method. **Figure 1B** demonstrates that SPC exhibited dose-dependent inhibition on the proliferation of *S. aureus*. The minimum inhibitory concentration (MIC) value of SPC was determined to be 7.8 mg/mL (crude drug concentration), and the minimum bactericidal concentration (MBC) value was 15.6 mg/mL (crude drug concentration). The chemical composition of SPC was characterized using UPLC-MS/MS (**Supplementary Figure S1**). Six compounds were identified in SPC by comparing the ion information and retention times between the multistage mass spectrometry fragments with the relevant information in the literature and databases (**Supplementary Table S2**). At a SPC concentration of 7.8 mg/mL, the concentrations of the six components were Chlorogenic acid: 7 μg/mL; Catechin: 45.4 μg/mL; Columbianetin: 1.2 μg/mL; Nodakenin: 5.2–156 μg/mL, Imperatorin: 15.5 μg/mL; Osthol: 37.4 μg/mL. Since the antimicrobial activity of SPC extracts may be the result of a combination of several components, the final choice in this study was to use the crude concentration of SPC as the MIC, i.e. 7.8 mg/mL. Several antibiotics are effective against *S. aureus*. So we chose ampicillin (AM) as a positive control drug and determined the MIC and MBC of AM against *S. aureus* using the same test method (**Supplementary Figure S2**), with values of 1 μg/mL (MIC) and 2 μg/mL (MBC), respectively.

Changes in bacterial morphology after SPC treatment can be observed by SEM and TEM. SEM images revealed that untreated *S. aureus* displayed a smooth surface with 1 μm in diameter (**Supplementary Figure S3**). However, after treatment with SPC, the bacteria exhibited irregular morphology and damaged cell walls. TEM images further confirmed these observations, showing that untreated *S. aureus* had uniform morphology, while *S. aureus* co-cultured with SPC exhibited transparent cells with blurred boundaries and cytoplasmic leakage (**Figure 1C**). SYTO 9 green fluorescent stain and PI red fluorescent stain are two common nucleic acid stains with different ability to penetrate bacteria. SYTO 9 stains all bacteria in a population regardless of cell membrane integrity. PI is a membrane-impermeable nucleic acid stain that can only penetrate bacteria with damaged cell membranes and is used to assess bacterial cell membrane integrity. As shown in **Figure 1D**, little red fluorescence was observed in the fluorescence images of the untreated group, in contrast, the intensity of red fluorescence was increased in *S. aureus* treated with SPC. This proved that most of the *S. aureus* in the control group was alive and the PI was blocked outside the cell membrane, whereas SPC was able to disrupt the cell membrane of *S. aureus*, leading to the production of red fluorescence.

### 3.2 Biofilm inhibition and eradication effect of SPC

**Figure 2:**
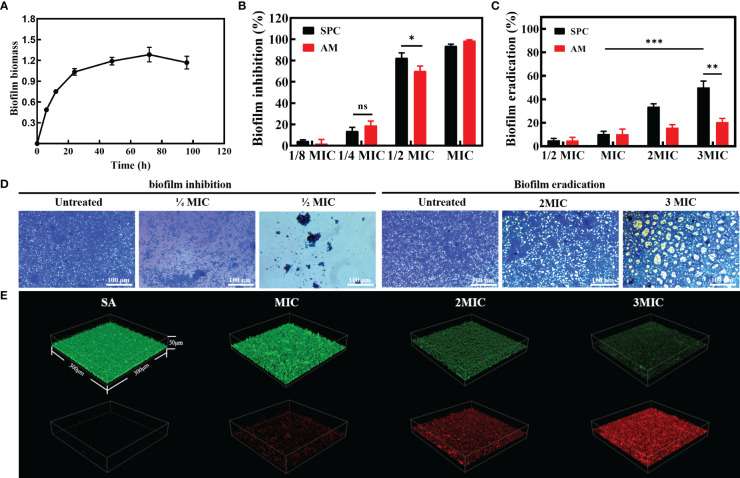
The efficacy of SPC in counteracting biofilm. **(A)** Biofilm formation ability of *S. Aureus*. (n = 3 per group). Data are presented as means ± SD for at least triplicate experiments. **(B)** Biofilm inhibition effect of SPC (n = 3 per group). Data are presented as means ± SD for at least triplicate experiments. (*p < 0.05; **p < 0.01; ***p < 0.001; n.s., not significant) **(C)** Biofilm eradication effect of SPC (n = 3 per group). Data are presented as means ± SD for at least triplicate experiments (*p < 0.05; **p < 0.01; ***p < 0.001). **(D)** Representative image of inhibition and eradication effects on bacterial biofilms after exposure to SPC (n=3 per group). Scale bar: 100 μm. **(E)** Representative images of 3D confocal laser scanning microscopy (CLSM) of mature biofilms after incubation with SPC (n=3 per group). The size of these image areas taken is 300 µm × 300 µm × 50 µm.

*S. aureus* is a pathogenic bacterium known for its remarkable ability to adapt to various environmental conditions, including high temperatures and high salt concentrations. In nature, *S. aureus* often forms biofilms, which are structured communities of bacterial cells enclosed in a self-produced matrix of extracellular polymeric substances (EPS). Considering that biofilm formation may increase the ability of *S. aureus* to resist external disturbances, we conducted an investigation to evaluate the inhibitory effect of SPC on biofilm formation. The formation ability of *S. aureus* biofilm was assessed using the Crystal violet staining method. As the culture time was extended, the absorbance steadily increased and reached its peak at 72 hours, indicating that the biofilm had fully matured after three days (**Figure 2A**). The control group displayed a deep purple color due to the interaction between crystal violet and negatively charged surface molecules in the extracellular matrix, implying the formation of a relatively complete biofilm (**Supplementary Figure S4**). However, the color of the SPC-treated group was noticeably lighter, revealing that SPC had the ability to inhibit biofilm formation. To quantify the biomass of the biofilms treated with various concentrations of SPC (ranging from 0 to MIC), crystal violet colorimetry was performed. The results, as shown in **Figure 2B**, demonstrated that SPC significantly reduced bacterial attachment in a dose-dependent manner. At 1/2 MIC, SPC exhibited a strong inhibition of biofilm formation, with an inhibition rate exceeding 80%. For mature biofilms, SPC demonstrated the ability to remove approximately 10.6% of the biofilm at MIC. As the concentration increased (up to 3 MIC), the biofilm clearance reached 50.2% (**Figure 2C**). Simultaneously, the same experiment was performed for AM and it was found that biofilm formation was almost completely inhibited at MIC concentrations, with 70.4% inhibition rate at 1/2MIC. In the biofilm removal ability, the performance of AM was greatly weakened. When the concentration of AM was increased to 3MIC, the removal rate of biofilm was only 20.5%, which may be due to the fact that AM was not able to penetrate into the extracellular matrix of the bacteria and could not play an effective role (**Figures 2B, C**). These findings hold promise for the eradication of *S. aureus* in diverse scenarios. To better visualize its effects, microscopic images were captured to support the biofilm inhibition and clearance of SPC. The results shown in **Figure 2D** align with the aforementioned conclusions. **Supplementary Figure S5** displayed microscopic images of the AM treatment group. To further analyze the impact of SPC on biofilm eradication, the average thickness of the biofilm formed on Petri dish surface was analyzed using 3D CLSM. The results, depicted in **Figure 2E**, showed that while *S. aureus* can easily form a certain thickness of biofilm on the surface of Petri dish, the biofilm in the SPC-treated group was much thinner. Simultaneously, the results obtained from fluorescent staining demonstrated that SPC exerted a concentration-dependent bactericidal effect on the bacteria within the biofilm. Additionally, we also examined the inhibition effect of SPC on biofilm formation by 3D CLSM and investigated the impact on the secretion of protein, which is the main component of *S. aureus* biofilms. **Supplementary Figure S6** displayed that the group treated with SPC could hardly form biofilms and **Supplementary Figure S7** revealed a significant reduction in the levels of protein after treatment with SPC, which was consistent with the inhibitory effect on biofilm formation.

### 3.3 Investigation of the potential mechanism of antibacterial of SPC

**Figure 3:**
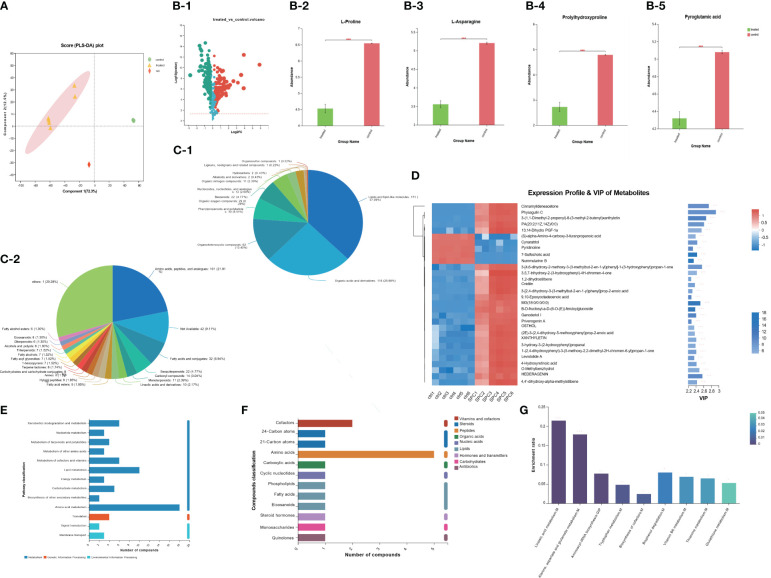
The result of S. aureus Metabolomics. **(A)** Partial Least Squares Discrimination Analysis (PLS-DA) model validation on identified metabolites. **(B)** Discrepant metabolites in comparison of *S. aureus* group (control group) and SPC group (treated group) (*p < 0.05; **p< 0.01; ***p< 0.001). **(C)** Pie chart based on counts of HMDB chemical taxonomy for all metabolites detected in this study class. **(D)** A heatmap of differentially expressed metabolites between the SPC and *S. aureus* groups, and the showing the top 30 significantly differentially expressed metabolites. **(E)** KEGG pathway classification of metabolites detected and annotated. **(F)** Compounds classification between the *S. aureus* group (control group) and SPC group (treated group) **(G)** The results of KEGG pathway enrichment analysis (*p < 0.05; **p < 0.01; ***p < 0.001).

The metabolite profiles of *S. aureus* samples were analyzed using LC-MS. Through this analysis, we identified a total of 267 differential metabolites in the SPC-treated group compared to the control group. The significance of these metabolites was determined using VIP based on the PCAs model (**Figures 3A, B**). Among the differential metabolites, the content of L-Proline, L-Asparagine, Prolylhydroxyproline, and Pyroglutamic acid in *S. aureus* metabolites was found to be higher in the control group than in the treated group (P-value<0.001, **Figure 3B**). The identified metabolites were further classified into different tiers based on the number of metabolites, as displayed in descending order (superclass shown in **Figure 3C1**, class shown in **Figure 3C2**). To gain a deeper understanding of the metabolic pathways affected by SPC, we utilized the KEGG pathway database, which describes molecular interactions, physiological and biochemical reactions, and relationships between gene and products. Three metabolic pathways were identified: Metabolism, Genetic Information Processing, and Environmental Information Processing. Among these, amino acid metabolism and lipid metabolism were the most abundant metabolites annotated in the classification of metabolism pathway (**Figure 3E**). Additionally, the identified metabolites were categorized into various groups based on their biological functions, such as organic acids, lipids, carbohydrates, nucleic acids, peptides, vitamins and cofactors, steroids, hormones and cofactors, and antibiotics (**Figure 3F**). KEGG pathway enrichment analysis revealed significant differences in the enrichment rates of Linoleic acid metabolism and Alanine, aspartate, and glutamate metabolism between the two groups (P-value<0.001, **Figure 3G**).

### 3.4 Exploration of the combined system of SPC and MOFs

**Figure 4:**
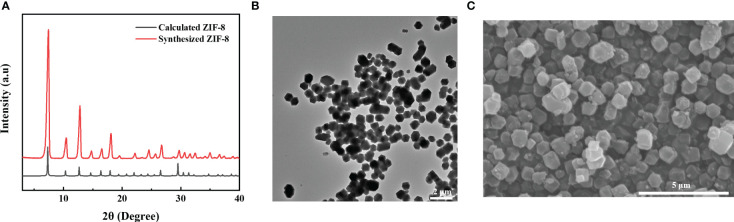
Physical characterization of MOFs. **(A)** PXRD patterns of ZIF-8. **(B)** TEM image of ZIF-8. Scale bar: 2 μm. **(C)** SEM image of ZIF-8. Scale bar: 5 μm.

The above experiments confirmed the weak inhibitory effect of SPC on *E. coli* (**Figure 1A**). Therefore, to overcome these limitations, we introduced MOFs with antibacterial effects. Three types of MOFs (ZIF-8, MIL-100(Fe) and MIL-101(Fe)) were selected as collaborative materials for SPC. The successful synthesis of ZIF-8, MIL-100(Fe) and MIL-101(Fe) was confirmed through PXRD, TEM, and SEM, which was in full agreement with previously reported results (**Figures 4A–C**; **Supplementary Figure S8**).

**Figure 5:**
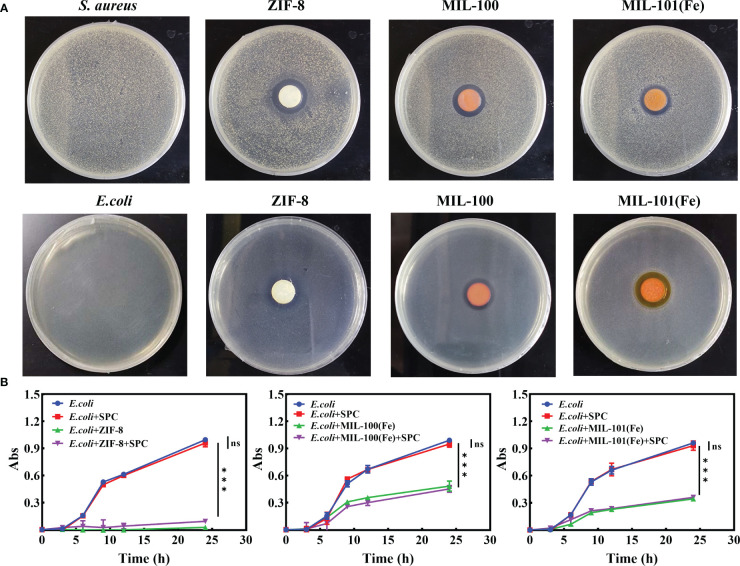
The combined effect of SPC and MOFs. **(A)** Representative image of inhibition zone of MOFs for *E*. *coli* and *S. Aureus* (n=3 per group). **(B)** Growth curve of *E*. *coli* treated with combination system (n=3 per group). Data are presented as means ± SD for at least triplicate experiments. (*p < 0.05; **p < 0.01; ***p < 0.001; n.s., not significant).

These MOFs’ effects on *E. coli* and *S. aureus* were tested using the agar disk diffusion method. The MOFs were sterilized, pressed into circular sheets, and placed in LB medium containing bacteria for culturing. The results, shown in **Figure 5A**, indicated that all three MOFs exhibited inhibitory effects on both *E. coli* and *S. aureus*. Additionally, the relative bactericidal activity was evaluated through bacterial growth kinetics, and the concentration suitable for the combination of SPC and MOFs was determined (**Supplementary Figure S9**). Furthermore, the effect of the combined system on *E. coli*, which was not sensitive to SPC, was tested. The combined system was prepared by simply mixing the appropriate concentration of SPC and MOFs. As depicted in **Figure 5B**, under the same treatment conditions, the mixed system had a significant effect on the growth kinetics of bacteria compared to the SPC group, indicating that the introduction of MOFs in SPC was helpful in expanding the antibacterial spectrum of SPC.

## 4 Discussion

*S. aureus* is a major human pathogen causing cardiovascular, skin, respiratory, surgical site and medical device infections. It is estimated that approximately 30% of people are asymptomatic nasal carriers of S. aureus (Chambers and DeLeo, 2009). A major problem associated with *S. aureus* is its ability to develop resistance to most antibiotics, including penicillin, methicillin and vancomycin (Cheung et al., 2021). Biofilms not only render drugs partially or completely ineffective by preventing or slowing their diffusion, but also have the ability to evade recognition by the body’s innate immunity, thus facilitating bacterial colonization of the body (Idrees et al., 2021). *S. aureus* is also able to form biofilms on non-living surfaces, particularly in healthcare facilities and food-related industries, posing a significant challenge to the prevention and control of hospital-acquired infections and food safety (Oniciuc et al., 2016; Tuon et al., 2023). Therefore, there is an urgent need to develop new therapeutic strategies for the treatment of drug resistance *S. aureus* and biofilm associated infections. Medicinal plants are one of the potential sources of natural compounds, and the use of TCM, which is a comprehensive exploration of the functions of a large number of medicinal plants, is well established in the treatment of infectious diseases. Therefore, the in-depth study of TCM prescriptions is a new direction in the search for novel antimicrobial drugs (Hu et al., 2022).

In this study, we investigated the *in vitro* antimicrobial activity of SPC extracts against *S. aureus*. MIC and MBC results showed a dose-dependent effect of SPC against *S. aureus*. TEM and live-dead cell staining further established the ability of SPC to disrupt bacterial cell permeability. The need for novel antibacterial agents to maintain good antibacterial efficacy against bacteria within biofilms emphasizes the importance of biofilm inhibition and clearance. The Crystal violet colorimetry assay showed that SPC significantly inhibited bacterial biofilm formation with a biofilm inhibition rate of over 80% at 0.5 MIC. In terms of biofilm clearance, SPC achieved 50.2% biofilm clearance at 3 MIC compared to 20.5% at 3 MIC for the positive control (ampicillin). In addition, laser confocal experiments on biofilms demonstrated the antibacterial effect of SPC on bacteria within the biofilm even after the formation of a mature biofilm, which was particularly significant in the treatment of chronic and hospital-acquired infections. These results suggest that SPC is an effective antibacterial agent against *S. aureus*. Metabolomics analyses allowed us to study the metabolites produced by *S. aureus* in the presence of SPC extracts and revealed the disruption of several metabolic pathways. Previous studies have also investigated the effect of herbs on *S. aureus* metabolomics, highlighting changes in key metabolites such as amino acids, fatty acids, nucleotides and cell wall components (Wang et al., 2019; Chen et al., 2020; Liu et al., 2023). This study further supports the potential of herbal extracts in combating opportunistic pathogens. The antimicrobial activity of TCM prescriptions is attributed to the participation of various active ingredients, such as flavonoids, alkaloids, and phenols (Li et al., 2022). SPC extracts contain multiple active components that can inhibit *S. aureus* proliferation through a variety of targets and pathways. We employed UPLC-MS/MS analysis to identify at least six compounds present in the SPC extract namely chlorogenic acid, catechin, columbianetin, nodakenin, imperatorin, osthol. These compounds exhibit a diverse range of biological effects. Chlorogenic acid from *Atractylodes lancea* has bacteriostatic and antibiofilm activity against Yersinia enterocolitica (Chen et al., 2022) and imperatorin from *Angelica dahurica* has antioxidant and anti-inflammatory properties (Nasser et al., 2019). Therefore, the analysis of the active components and the antimicrobial capacity of SPC extracts offers a series of advantages for the development of new drugs against opportunistic pathogens and for finding alternative therapeutic options. Firstly, the analysis of SPC extracts facilitates the discovery of one or more new antimicrobial active compounds. Secondly, the antimicrobial activity of SPC extracts is a result of the association of different components, which provides the basis for the precise screening of the effective combinations of different active components. Furthermore, as the advances in the extraction technology of active ingredients and the development of network pharmacology, TCM prescriptions extracts provide a large number of potential antimicrobial candidates.

Antimicrobial resistance has emerged as a pressing issue due to the chronic abuse of antibiotics. Moreover, relying on a single antibacterial mechanism often results in limited efficacy. To overcome this challenge, we integrated MOFs into SPC to achieve a combined antibacterial effect through multiple mechanisms (Pettinari et al., 2021; Han et al., 2022). In general, MOFs possess several antimicrobial mechanisms: (1) MOFs can be constructed by bactericidal metal ions and/or organic antibacterial agents. Under specific environments (such as pH variations), they can release their components in a controlled manner, allowing for targeted antibacterial effects. (2) The high porosity and specific surface area of MOFs facilitate the efficient loading of various antibacterial drugs, leading to synergistic effects. (3) MOFs that exhibit outstanding photocatalytic performance can generate reactive oxygen species, achieving a photocatalytic antibacterial effect. (4) The inherent properties of MOFs, including unique morphology, surface modifications, and size, make them favorable for the adhesion and penetration of bacteria. The combination of herbal extracts and MOFs represents a new approach against bacterial antibiotic resistance. Furthermore, MOFs can serve as substrate materials for various application scenarios, including nanoformulations, and masks (Qiao et al., 2020; Duan et al., 2021; Zhang et al., 2023). This opens up new possibilities for the application forms of herbal extracts. To evaluate the antibacterial activity of MOFs in combination with SPC extracts, various scientific studies including MOFs synthesis, compatibility testing, and efficacy evaluation were conducted. The results showed that MOFs were compatible with SPC and effectively overcame the weak inhibitory effect of SPC on *E. coli*. This means that the use of MOFs can prevent bacterial contamination (*E. coli*) that may occur during the storage stage of SPC and expand its antibacterial spectrum.

## 5 Conclusion

In this study, we have demonstrated that SPC extracts can effectively inhibit the metabolic proliferation of *S. aureus* through multiple targets and pathways. The addition of MOFs to the SPC system successfully broadened the antibacterial spectrum. However, further research is necessary to investigate the effectiveness, safety, and specific applications of SPC herbal medicine combined with MOFs as an antimicrobial agent, including the study of specific extracts, the analysis of active ingredients, the combination form of SPC and MOFs, and the compatibility with different formulations (Han et al., 2018; Huang et al., 2020; Kumar et al., 2023). In conclusion, both TCM and MOFs have great prospects in the field of antibacterial research. The convergence of these two fields can drive the development of new antibacterial drugs, overcome the challenge of drug-resistant bacteria, and provide new solutions for antibacterial treatment. Further investigations and collaborations between TCM and materials science are essential in exploring and harnessing the benefits of this combination (Guan et al., 2021; Zhang et al., 2021).

## Data availability statement

The original contributions presented in the study are included in the article/**Supplementary Material**. Further inquiries can be directed to the corresponding authors.

## Author contributions

HJ: Conceptualization, Data curation, Investigation, Methodology, Validation, Writing – original draft, Writing – review & editing. XZ: Investigation, Methodology, Writing – original draft. XQM: Formal analysis, Visualization, Writing – review & editing. XM: Investigation, Resources, Writing – review & editing. ZL: Funding acquisition, Resources, Writing – review & editing. XL: Supervision, Writing – review & editing, Conceptualization, Funding acquisition, Project administration, Resources, Writing – original draft. GH: Supervision, Writing – review & editing, Validation. YC: Conceptualization, Resources, Writing – review & editing, Writing – original draft.
