# Supplementary material for: Utilization of the Shensheng-Piwen changed medicinal powder extracts combines metal-organic frameworks as an antibacterial agent
Source: Front Cell Infect Microbiol. 2024 Jun 7;14:1376312. doi: 10.3389/fcimb.2024.1376312 (PMC11193333; doi:10.3389/fcimb.2024.1376312)
Supplement: Supplementary file 1 [file DataSheet_1.docx]

Supplementary Material

Utilization of the SPC Herbal Medicine ethanol product of Heated Distillation combines MOFs as an Antimicrobial Agent

# Haiqun Jin^2^, Xiujun Zhang^3^, Xiaoqing Ma^4^, Xin Meng^6^, Zhenguang Lin^7^, Guojie Hu^1^*, Xiaoyuan Li1,^5^*, Yao Chen^2^

# ^1^ Department of Traditional Chinese Medicine, Affiliated Hospital of Qingdao University, Qingdao, China

# ^2^ State Key Laboratory of Medicinal Chemical Biology, Nankai University, Tianjin, Shandong Academy of Pharmaceutical Sciences, Jinan, Haihe Laboratory of Synthetic Biology Tianjin, China

# ^3^ Tianjin Academy of Traditional Chinese Medicine Affiliated Hospital, Tianjin, Shandong Academy of Pharmaceutical Sciences, Jinan, China

# ^4^ Key Laboratory of Marine Drugs, Ministry of Education, School of Medicine and Pharmacy, Ocean University of China, Qingdao, Marine Biomedical Research Institute of Qingdao, Qingdao, China

# ^5^ College of Materials Science and Engineering, Qingdao University, Qingdao, China

# ^6^ Key Laboratory of Industrial Microbiology, Ministry of Education, College of Biotechnology, Tianjin University of Science and Technology, Tianjin, Shandong Academy of Pharmaceutical Sciences, Jinan, China

# ^7^ Shandong Academy of Pharmaceutical Sciences, Jinan, China

# Supplementary Data

## Extracellular protein production inhibition assay

To perform the experiment, a sterile cover glass, S. aureus bacterial strain, and SPC at different concentrations were added to the 24-well plate and incubated at 37 ℃ for 3 days to allow biofilm formation. After the incubation period, the medium was discarded and the unattached bacteria were washed with sterile PBS. The cover glass was then placed in PBS and subjected to ultrasound treatment for 30 minutes to disrupt the biofilm and release its components into the solution. Finally, the solution was centrifuged at 10,000rpm for 10 minutes, and the supernatant was collected as the extracellular polymeric substances (EPS) sample for further analysis. The protein content of the EPS samples was measured by a BCA protein detection kit.

## UPLC- QE- Orbitrap-MS Methods of SPC

Chromatographic column: Infinity Lab Poroshell 120EC-C18 (150×2.1mm, 2.7μm) column, mobile phase: 0.1% formic acid water (A) - acetonitrile (B)；Flow rate: 0.2 mL-min-1；Injection volume: 2 µL; Column temperature: 35 ℃; Elution method: 0~5 min, 10% B; 5~10 min, 10%-21% B; 10~20 min, 21% B; 20~30 min, 21%-70% B; 30~35 min, 70% B.

Mass spectrometry conditions: positive and negative ion mode detection, sheath gas flow rate 40 psi (1 psi = 6.895 kPa), auxiliary gas flow rate 10 psi; spray voltage 3.8 kV; capillary temperature 320°C; auxiliary gas temperature 350°C; mass scan range m/z 100-1500, detection resolution 70,000 FWHM. Ionization energies 20, 30, 40 eV, resolution 17500.

## Metabolomic Methods

### Metabolite Extraction

Liquid sample：100μl liquid sample were extracted using a 400 µL methanol:acetonitrile (1:1, v/v) solution. The mixture then sonicated at 40 kHz for 30 min at 5oC. The samples were placed at -20oC for 30min to precipitate proteins. After centrifugation at 13000g at 4oC for 15min, the supernatant were carefully transferred to new microtubes and evaporated to dryness under a gentle stream of nitrogen. For UPLC-MS/MS anlaysis, the samples were reconstituted in 100 µL loading solution of acetonitrile :water (1:1, v/v) by brief sonication in a 5oC water bath. Extracted metabolites were spun for 15 min at 13000g at 4oC on a bench-top centrifuge and cleared supernatant were transferred to sample vials for LC-MS/MS analysis

### Quality control sample

As a part of the system conditioning and quality control process, a pooled quality control sample (QC) was prepared by mixing equal volumes of all samples. The QC samples were disposed and tested in the same manner as the analytic samples. It helped to represent the whole sample set, which would be injected at regular intervals (every 10 samples) in order to monitor the stability of the analysis.

### UHPLC-MS/MS analysis.

The instrument platform for this LC-MS analysis is UHPLC-Q Exactive HF-X system of Thermo Fisher Scientific.

Chromatographic conditions：2μL of sample was separated by HSS T3 column (100 mm × 2.1 mm i.d., 1.8 μm) and then entered into mass spectrometry detection.The mobile phases consisted of 0.1% formic acid in water:acetonitrile (95:5, v/v) (solvent A) and 0.1% formic acid in acetonitrile:isopropanol:water (47.5:47.5:5, v/v)(solvent B). The solvent gradient changed according to the following conditions: from 0 to 3.5 min, 0% B to 24.5% B (0.4 mL/min); from 3.5 to 5 min, 24.5% B to 65% B (0.4 mL/min); from 5 to 5.5 min, 65% B to 100% B (0.4 mL/min); from 5.5to 7.4 min, 100% B to 100% B (0.4 mL/min to 0.6 mL/min); from 7.4 to 7.6 min, 100% B to 51.5% B (0.6 mL/min); from 7.6 to 7.8 min, 51.5% B to 0% B (0.6 mL/min to 0.5 mL/min); from 7.8 to 9 min, 0% B to 0% B (0.5 mL/min to 0.4 mL/min);from 9 to 10 min, 0% B to 0% B (0.4 mL/min) for equilibrating the systems. The sample injection volume was 2 µL and the flow rate was set to 0.4 mL/min. The column temperature was maintained at 40 oC. During the period of analysis, all these samples were stored at 4 oC.

MS conditions: the mass spectrometric data was collected using a Thermo UHPLC -Q Exactive HF-X Mass Spectrometer equipped with an electrospray ionization (ESI) source operating in either positive or negative ion mode. The optimal conditions were set as followed: heater temperature, 425 oC ; Capillary temperature, 325 oC; sheath gas flow rate, 50 arb; Aux gas flow rate, 13 arb; ion-spray voltage floating (ISVF),-3500V in negative mode and 3500V in positive mode, respectively; Normalized collision energy , 20-40-60V rolling for MS/MS. Full MS resolution was 60000, and MS/MS resolution was 7500. Data acquisition was performed with the Data Dependent Acquisition (DDA) mode. The detection was carried out over a mass range of 70-1050 m/z.

### Data preprocessing and annotation

After the mass spectrometry detection is completed, the raw data of LC/MS is preprocessed by Progenesis QI (Waters Corporation，Milford, USA) software, and a three-dimensional data matrix in CSV format is exported. The information in this three-dimensional matrix includes: sample information, metabolite name and mass spectral response intensity. Internal standard peaks, as well as any known false positive peaks (including noise, column bleed, and derivatized reagent peaks), were removed from the data matrix, deredundant and peak pooled. At the same time, the metabolites were searched and identified, and the main database was the HMDB(http://www.hmdb.ca/), Metlin( https://metlin.scripps.edu/) and Majorbio Database .

Metabolic features detected at least 80 % in any set of samples were retained. After filtering, minimum metabolite values were imputed for specific samples in which the metabolite levels fell below the lower limit of quantitation and each Metabolic features were normalized by sum. In order to reduce the errors caused by sample preparation and instrument instability, the response intensity of the sample mass spectrum peaks was normalized by the sum normalization method, and the normalized data matrix was obtained. At the same time, variables with relative standard deviation (RSD) > 30% of QC samples were removed, and log10 logarithmization was performed to obtain the final data matrix for subsequent analysis.

### Differential metabolites analysis

Perform variance analysis on the matrix file after data preprocessing. The R package ropls (Version 1.6.2) performed principal component analysis (PCA) and orthogonal least partial squares discriminant analysis (OPLS-DA), and used 7-cycle interactive validation to evaluate the stability of the model. In addition, student's t-test and fold difference analysis were performed. The selection of significantly different metabolites was determined based on the Variable importance in the projeciton (VIP) obtained by the OPLS-DA model and the p-value of student’s t test, and the metabolites with VIP>1, p<0.05 were significantly different metabolites. A total of 267 differential metabolites were screened.

Differential metabolites among two groups were summarized , and mapped into their biochemical pathways through metabolic enrichment and pathway analysis based on database search (KEGG, http://www. genome.jp/kegg/). These metabolites can be classified according to the pathways they involved or the functions they performed. Enrichment analysis was usually to analyze a group of metabolites in a function node whether appears or not. The principle was that the annotation analysis of a single metabolite develops into an annotation analysis of a group of metabolites. scipy.stats (Python packages) ( https://docs.scipy.org/doc/scipy/ ) was exploited to identify statistically significantly enriched pathway using Fisher’s exact test.

# Supplementary Figures and Tables

## Supplementary Tables

**Supplementary Table 1.** Chinese medicinal herb contained in Shensheng-Piwen changed medicinal powder.

| Botanical plant name | Btanical taxonomy | Chinese name | Amount | batch number | Manufacturer source |
| --- | --- | --- | --- | --- | --- |
| Atractylodes lancea (Thunb.) DC. | Atractylodes | Cang Zhu | 5g | 201201 | Anhui Jingcheng Herbal decoction pieces LTD, Ltd., Co. |
| Hansenia weberbaueriana (Fedde ex H. Wolff) Pimenov & Kljuykov | Hansenia | Qiang Huo | 5g | 151101 | Qingdao Shanda Tianyuan Chinese Medicine slices LTD, Ltd., Co. |
| Heracleum hemsleyanum Diels | Heracleum | Du Huo | 5g | 171201 | Qingdao Shanda Tianyuan Chinese Medicine slices LTD, Ltd., Co. |
| Angelica dahurica (Fisch. ex Hoffm.) Benth. & Hook. f. ex Franch. & Sav. | Angelica | Bai Zhi | 5g | 170901 | Qingdao Shanda Tianyuan Chinese Medicine slices LTD, Ltd., Co. |
| Rhizoma Cyperi | Cyperus | Xiang Fu | 5g | 171001 | Qingdao Shanda Tianyuan Chinese Medicine slices LTD, Ltd., Co. |
| Folium Artemisiae Argyi | Compositae | Ai Ye | 5g | 170201/160301 | Qingdao Shanda Tianyuan Chinese Medicine slices LTD, Ltd., Co. |
| Conioselinum anthriscoides (H. Boissieu) Pimenov & Kljuykov | Conioselinum | Gao Ben | 5g | 161101/170801 | Linqu Pharmaceutical LTD, Ltd., Co. |
| Radix et Rhizoma Rhei | Polygonaceae | Da Huang | 5g | 180301/1707001 | Qingdao Shanda Tianyuan Chinese Medicine slices LTD, Ltd., Co. |
| Nardostachys jatamansi (D. Don) DC. | Nardostachys | Gan Song | 5g | 19120502/180501 | ShangPharma Holdings Qingdao LTD, Ltd., Co. |
| Ramulus Euonymi | Celastraceae | Gui Jian Yu | 5g | 180101 | Linqu Pharmaceutical LTD, Ltd., Co. |

**Supplementary Table 2.** The representative 6 components of SPC were identified by UPLC-MS/MS analysis.

| Compound | | Apex RT (min) | Area | Concentration (mg/g) |
| --- | --- | --- | --- | --- |
| 1 | Chlorogenic acid | 4.829 | 7276798744 | 0.9 |
| 2 | Catechin | 5.177 | 7641942044 | 5.82 |
| 3 | Columbianetin | 23.142 | 2393320694 | 0.16 |
| 4 | Nodakenin | 30.679 | 3346809005 | 0.661～20.059 |
| 5 | Imperatorin | 41.666 | 1039393432 | 1.99 |
| 6 | Osthol | 42.646 | 9718190255 | 4.8 |

## Supplementary Figures


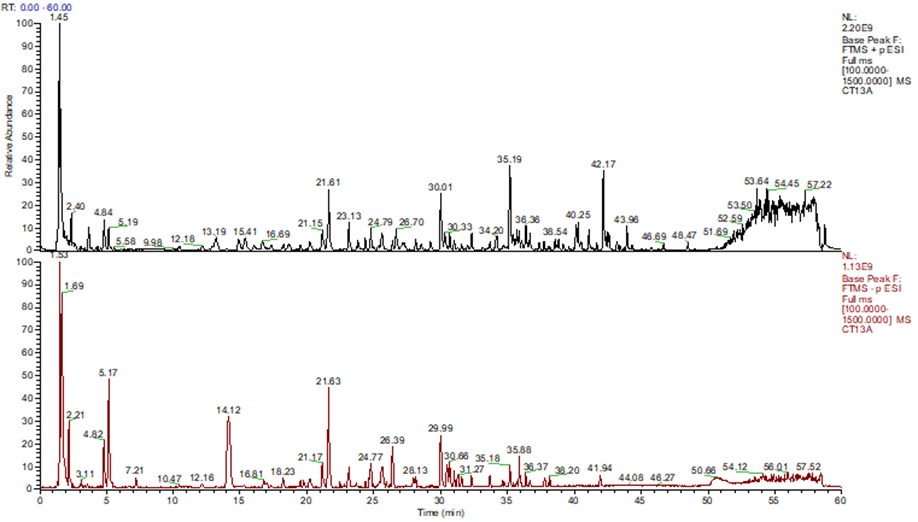


**Supplementary Figure 1.** UPLC-QE- Orbitrap-MS of SPC

**
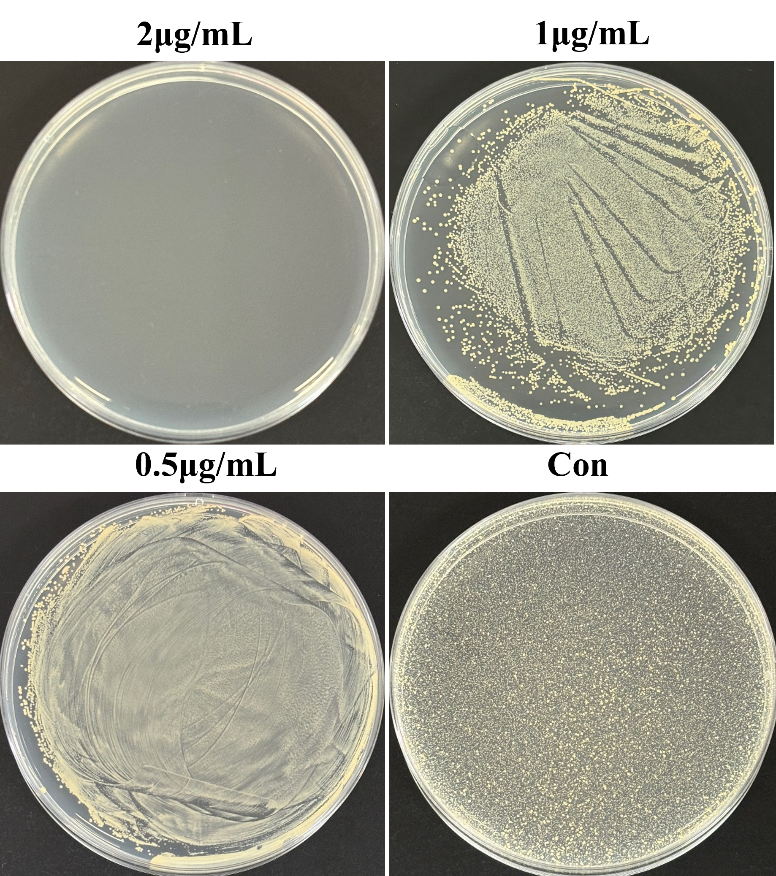
**

**Supplementary Figure 2.** Amount of live bacteria of *S. aureus* treated with different concentrations of AM (n=3 per group).


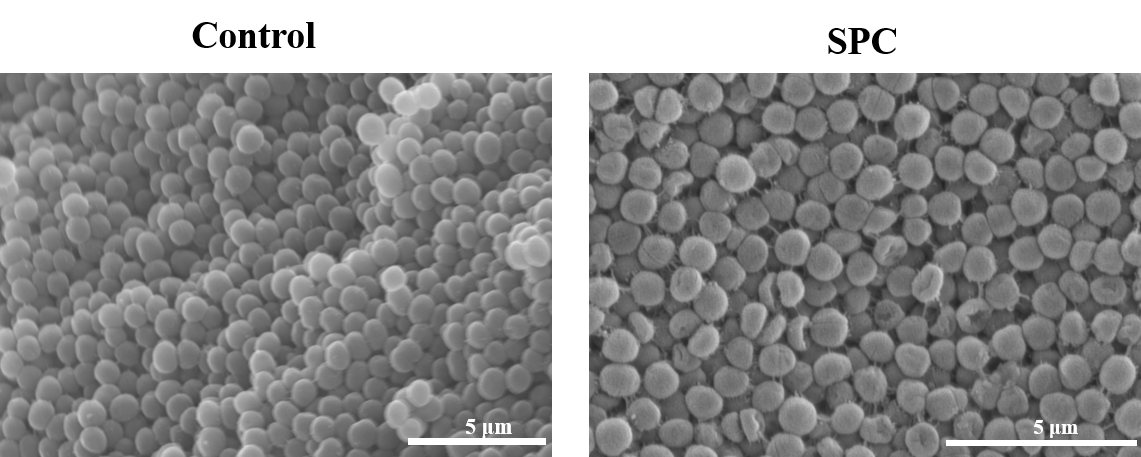


**Supplementary Figure 3.** Representative SEM image of *S. aureus* (n=3 per group). Scale bar: 5 μm.

.
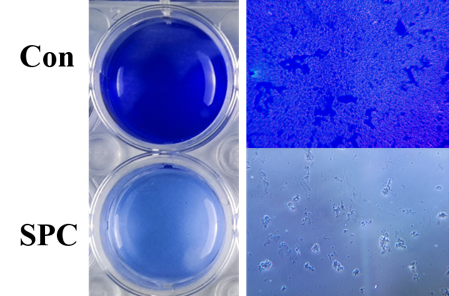


**Supplementary Figure 4.** Representative image of crystal-violet-stained biofilm dissolved in 33% glacial acetic acid solution in a 24-well plate (n=3 per group).


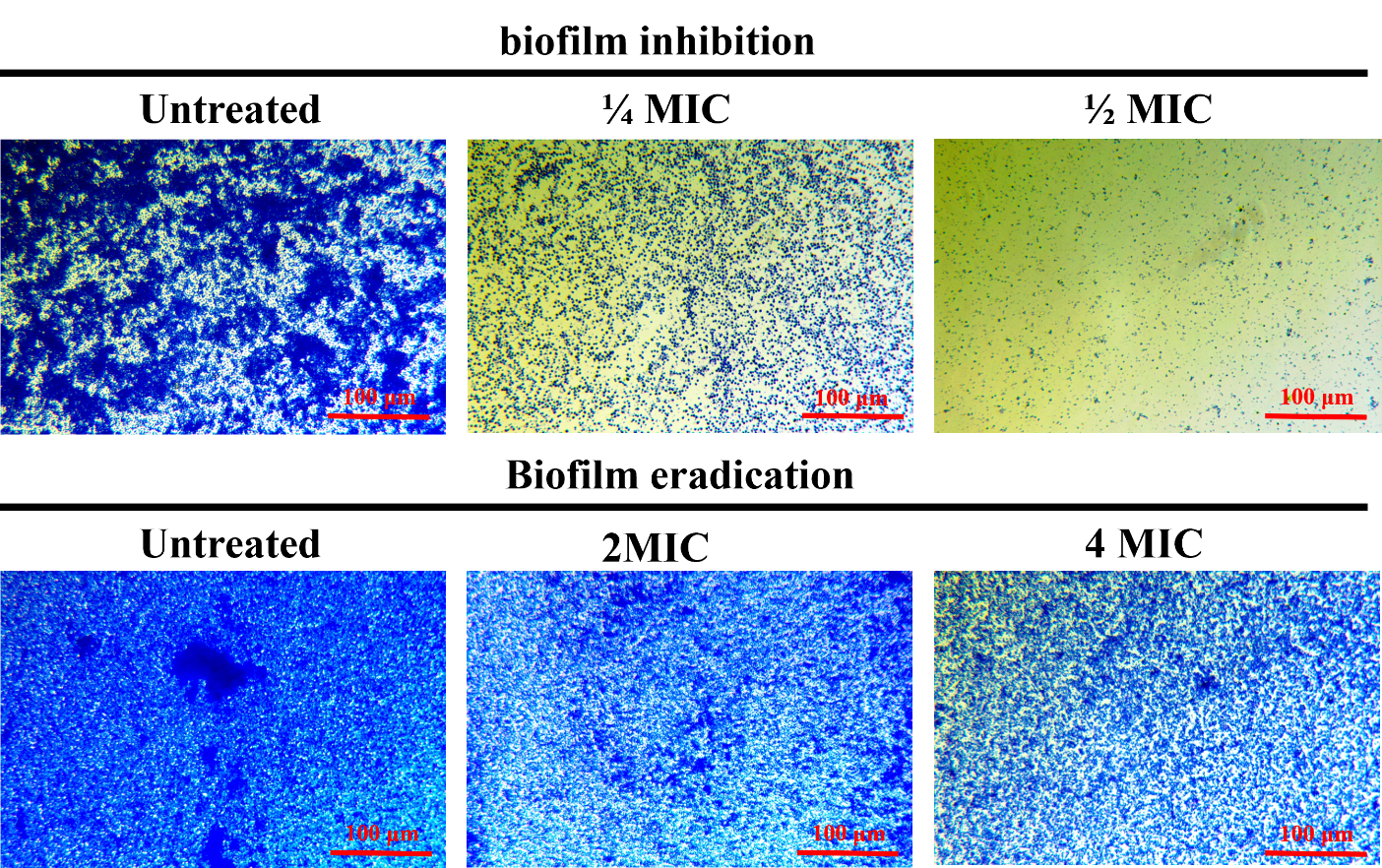


**Supplementary Figure 5.** Representative image of inhibition and eradication effects on bacterial biofilms after exposure to SPC (n=3 per group). Scale bar: 100 μm.


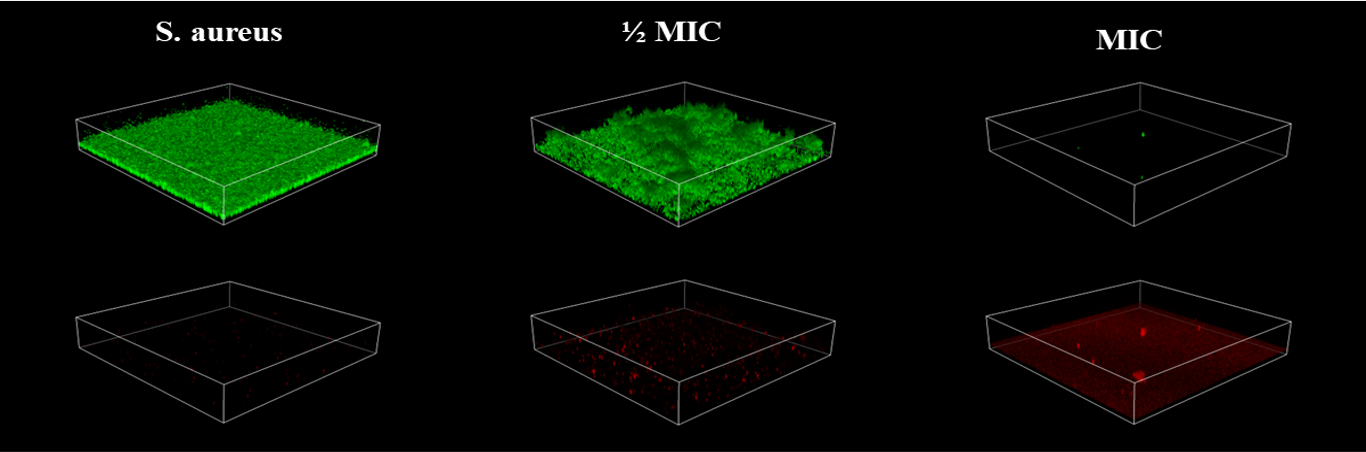


**Supplementary Figure 6.** Representative images of 3D confocal laser scanning microscopy (CLSM) of biofilm after incubation with SPC (n=3 per group). Live bacteria and dead bacteria presented green and red fluorescence under CLSM, respectively.


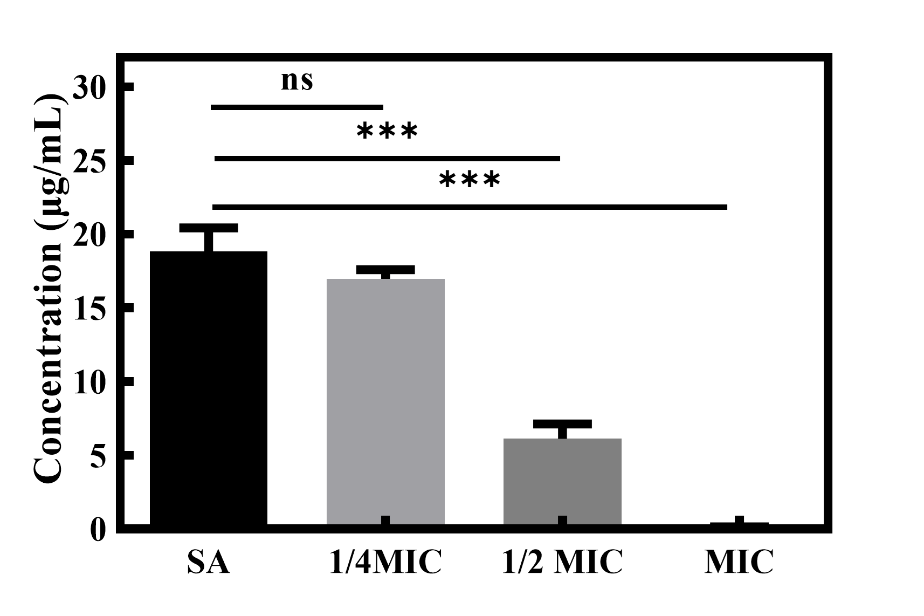


**Supplementary Figure 7.** Effect of SPC on extracellular protein of *S. aureus* biofilm. Data are presented as means ± SD for at least triplicate experiments. (*p < 0.05; **p< 0.01; ***p< 0.001; n.s., not significant).


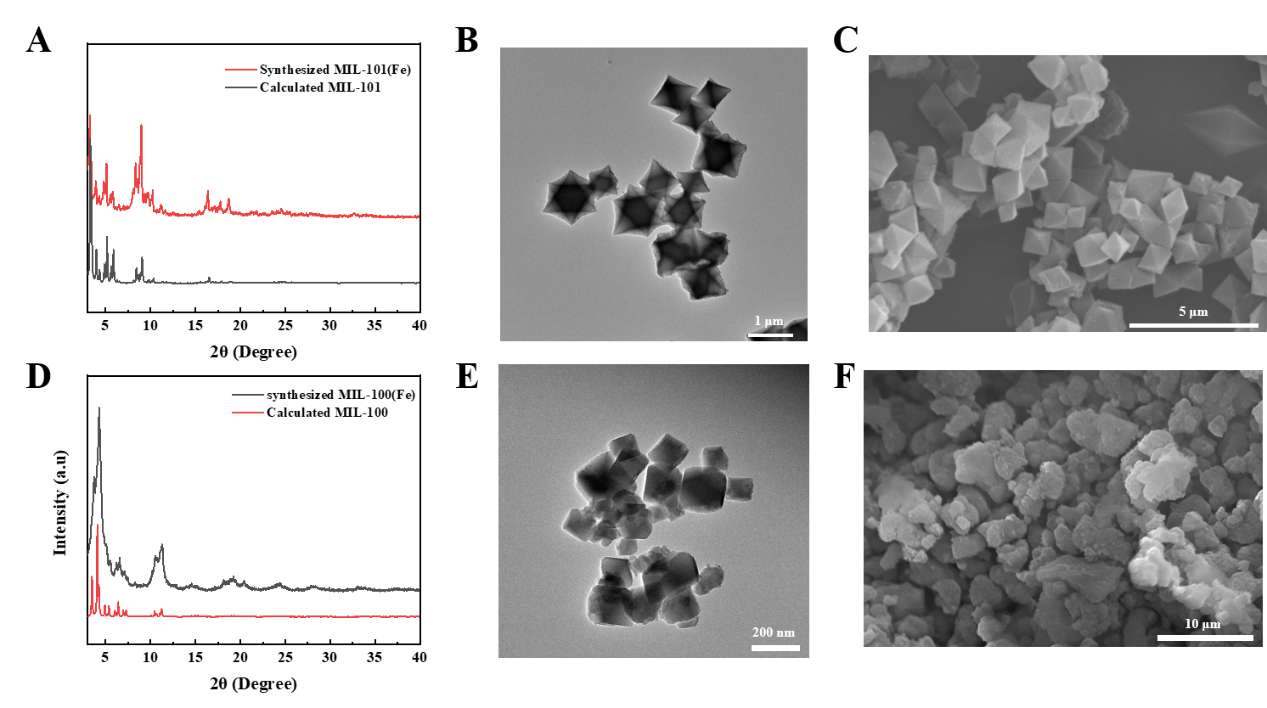


**Supplementary Figure8. (**A, D) PXRD patterns of MIL-101(Fe) and MIL-100(Fe). (B, E) TEM image of MIL-101(Fe) and MIL-100(Fe). (C, F) SEM image of MIL-101(Fe) and MIL-100(Fe).


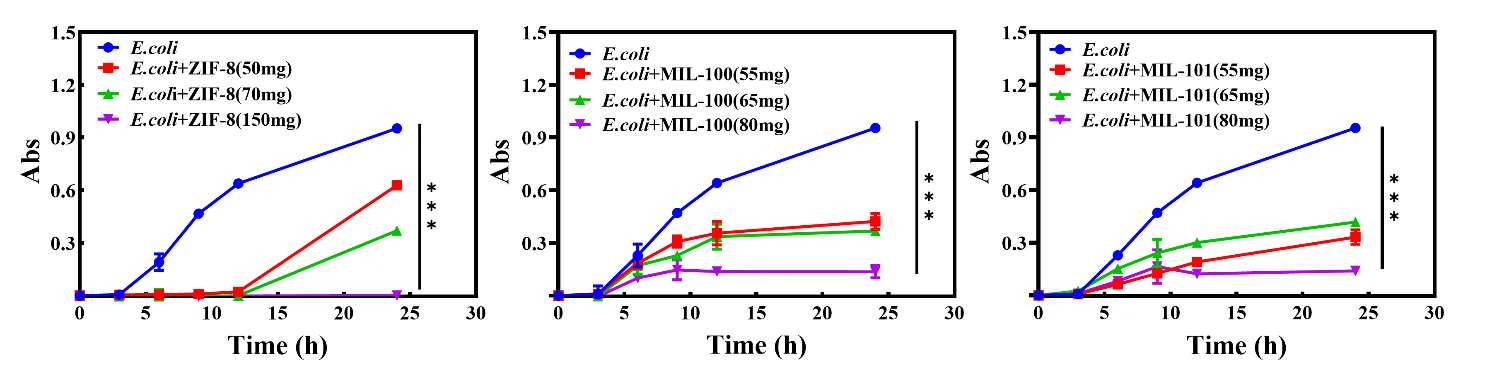


**Supplementary Figure 9.** Effects of different concentrations of MOFs on the growth of *E. coli*. Data are presented as means ± SD for at least triplicate experiments. (*p < 0.05; **p< 0.01; ***p< 0.001; n.s., not significant).
